# Developmental Differences in the Relationships Between Sensorimotor and Executive Functions

**DOI:** 10.3389/fnhum.2021.714828

**Published:** 2021-08-12

**Authors:** Chloe Gordon-Murer, Tino Stöckel, Michael Sera, Charmayne M. L. Hughes

**Affiliations:** ^1^Health Equity Institute, San Francisco, CA, United States; ^2^Department of Kinesiology, San Francisco State University, San Francisco, CA, United States; ^3^Sport & Exercise Psychology Unit, Department of Sport Science, University of Rostock, Rostock, Germany

**Keywords:** cognitive-motor interaction, executive functions, sensorimotor functions, eye-hand coordination, proprioceptive acuity, child development, children, adolescents

## Abstract

**Background:**

There is evidence that sensorimotor and executive functions are inherently intertwined, but that the relationship between these functions differ depending on an individual’s stage in development (e.g., childhood, adolescence, adulthood).

**Objective:**

In this study, sensorimotor and executive function performance was examined in a group of children (*n* = 40; 8–12 years), adolescents (*n* = 39; 13–17 years), and young adults (*n* = 83; 18–24 years) to investigate maturation of these functions, and how the relationships between these functions differ between groups.

**Results:**

Adults and adolescents outperformed children on all sensorimotor and executive functions. Adults and adolescents exhibited similar levels of executive functioning, but adults outperformed adolescents on two sensorimotor functioning measures (eye-hand coordination spatial precision and proprioceptive variability). Regression analysis demonstrated that executive functions contribute to children’s sensorimotor performance, but do not contribute to adolescent’s sensorimotor performance.

**Conclusion:**

These findings highlight the key role that developmental stage plays in the relationship between sensorimotor and executive functions. Specifically, executive functions appear to contribute to more successful sensorimotor function performance in childhood, but not during adolescence. It is likely that sensorimotor functions begin to develop independently from executive functions during adolescence, and therefore do not contribute to successful sensorimotor performance. The change in the relationship between sensorimotor and executive functions is important to take into consideration when developing sensorimotor and executive function interventions.

## Introduction

Imagine you are in a crowded market trying to get from one vendor to another, constantly needing to adjust the route you are taking in order to avoid bumping into others. Although this common task may seem effortless and simple, errorless performance requires that humans engage both sensorimotor and cognitive systems throughout all phases of the task. The acquisition and integration of sensorimotor and cognitive functioning processes is not an innate characteristic of humans, rather there is now consensus that these functions improve and develop with age throughout childhood and into adolescence (Cignetti et al., 2016; Morita et al., 2016).

While there is some variability as to the specific time period at which cognitive functions reach maturity (i.e., adult levels of performance) it is generally accepted that executive functions (EF, a specific subset of cognitive behavioral control), exhibit protracted developmental timelines (cf. Diamond, 2013) approaching maturity in early adolescence, and typically reach maturation by mid adolescence (Farrell Pagulayan et al., 2006; Luciana et al., 2009; Dajani and Uddin, 2015). For example, it has been reported that cognitive flexibility performance reaches adult-like levels between the ages of 10 and 12 years (Dajani and Uddin, 2015), while working memory has been found to reach maturation by 14 years of age (Farrell Pagulayan et al., 2006). Similar observations are found for sensorimotor functions, with studies reporting that 10 years olds demonstrate adult-like anticipatory motor planning consistency when using a dichotomous task (Wunsch et al., 2015), and that wrist proprioceptive acuity reaches maturation by the age of 12 (Marini et al., 2017b). Additionally, there is some evidence to support small improvements in executive functioning into early adulthood (e.g., over 18 years of age; Satterthwaite et al., 2013; Taylor et al., 2015) that is influenced by both biological and environmental changes occurring in early adulthood (Friedman et al., 2016).

The similar protracted developmental timelines of specific sensorimotor functions and EFs suggest that there are interrelations between these functions. Findings from recent studies (Roebers and Kauer, 2009; Weigelt et al., 2009; Stöckel and Hughes, 2016; Stöckel et al., 2017; Oberer et al., 2018; Stuhr et al., 2020), have indicated that sensorimotor functions and EFs are inherently intertwined. For example, Weigelt et al. (2009) found a link between working memory and perceptual-motor performance. Additionally, Stöckel et al. (2017) found weak to moderate associations between motor planning and multiple EFs including inhibitory control, cognitive flexibility, and planning and problem solving in a group of younger and older adults.

However, it appears that the relations between these functions differ depending on developmental stage (van der Fels et al., 2015; Stuhr et al., 2020), with stronger connections found in younger children than in adults. Specifically, Stuhr et al. (2020) found moderate to strong associations between working memory and three motor functions (strength, speed, and manual dexterity) in preschool children, but no significant associations between any measured cognitive and motor functions in young adults. Additionally, Stuhr et al. (2020) reported that working memory explained unique portions of preschool children’s speed and manual dexterity. Furthering this line of research, van der Fels et al. (2015) proposed that the connection between sensorimotor functions and EFs are stronger in childhood than in adolescence because of the changes in the developmental rate of these functions. Specifically, van der Fels et al. (2015) suggest that during childhood sensorimotor functions and EFs develop rapidly and with parallel trajectories, whereas the rate of development in adolescence differ and have divergent trajectories. This notion is supported by findings indicating that manual dexterity is associated with working memory and inhibitory control, and motor planning is associated with working memory and planning and problem-solving abilities in preschool children (5–6 years of age, Stöckel and Hughes, 2016), while in adolescent children (12–16 years of age, Rigoli et al., 2012) working memory is associated with aiming and catching performance, but not manual dexterity or balance.

At present, no study has examined the relations between these functions in child, adolescent, and adult groups. Motivated by this gap in the literature, the primary aim of the present study was to assess how children, adolescents, and adults differ in their performance of sensorimotor functions and EFs, with adults representing mature performance (i.e., upper bound performance). The secondary aim was to explore the specific links between sensorimotor functions and EFs, and how these differ during childhood, adolescents and adulthood. To this end, 40 children (8–12 years old), 39 adolescents (13–17 years old), and 83 young adults (18–24 years old), all neurologically and physically healthy, performed a battery of sensorimotor and EF tasks that measure anticipatory motor planning (Grasp Height Task), proprioceptive acuity (Ipsilateral Joint Position Matching Task), eye hand coordination (Pursuit Rotor Task), planning and problem-solving (Tower of London Task), cognitive flexibility (Wisconsin Card Sorting Task), working memory (Corsi Block-Tapping Task), and inhibitory control (Simon Task). Based on previous work, we expected that adults would outperform children on all measures (Davol et al., 1965; Farrell Pagulayan et al., 2006; Luciana et al., 2009; Dajani and Uddin, 2015; Wunsch et al., 2016; Marini et al.,2017a,b; Stuhr et al., 2020). Additionally, we hypothesized that the associations between sensorimotor functions and EFs would be stronger in childhood than in adolescence and adulthood (van der Fels et al., 2015; Stuhr et al., 2020) due to the crucial role that EFs play in sensorimotor performance during childhood.

## Materials and Methods

### Participants

Forty typically developing children (age range = 8–12 years, mean age = 10.1 ± 1.5 years, 18 females), 39 typically developing adolescents (age range = 13–17 years, mean age = 15.2 ± 1.3 years, 18 females), and 83 young adults (age range = 18–24 years, mean age = 22.1 ± 1.4 years, 41 females) participated in this study (see Table 1). All participants had normal or corrected to normal vision, and normal hearing. Participants were excluded if they had any known neurological or physical disorders that could impair their ability to perform activities of daily living, had an Individualized Education Plan (IEP), or were unable to speak and understand English. The experiment, and informed consent and assent were approved by the San Francisco State University Institutional Review Board.

**TABLE 1:** Demographic characteristics, descriptive statistics on raw data [means and standard deviation (in parentheses)], as well as results of one way ANOVAs (on the normalized data) used to examine differences in sensorimotor and cognitive functioning between children, adolescents, and young adults.

	Children (*n* = 40)	Adolescents (*n* = 39)	Adults (*n* = 83)	*p*	*F*	η^2^
Age in years	10.1 (1.5)	15.2 (1.3)	22.1 (1.4)	–	–	–
Females (%)	18 (45.0)	18 (46.2)	41 (49.4)	–	–	–
Right hand dominant (%)	39 (97.5)	36 (92.3)	76 (90.6)	–	–	–
**Sensorimotor functions**						
Anticipatory motor planning, number of successful trials (AMP)	3.70 (2.0)*	5.44 (1.90)	5.53 (1.78)	<0.001	13.46	0.145
Eye-hand coordination accuracy, mean time-on-target (%, EH_Acc_)	42.6 (15.9)*	64.4 (10.46)	66.2 (15.04)	<0.001	35.04	0.306
Eye-hand coordination spatial precision, linearity index (EH_SP_)	1.35 (0.21)*	1.15 (0.08)*	1.11 (0.09)*	<0.001	36.26	0.313
Proprioceptive acuity, matching error (°, P_ME_)	7.23 (3.0)*	5.21 (1.89)	5.23 (1.85)	<0.001	10.22	0.114
Proprioceptive acuity, variability (°, P_Var_)	6.89 (3.6)*	4.75 (1.79)*	4.04 (1.24)*	<0.001	20.36	0.204
**Executive functions**						
Planning and problem-solving, successful trials (%, ToL)	54.4 (16.9)*	75.2 (18.8)	77.3 (15.0)	<0.001	23.98	0.232
Cognitive flexibility, correct trials (%, WCST)	73.7 (12.4)*	81.4 (9.1)	81.3 (8.07)	<0.001	10.61	0.118
Working memory, Corsi product (Corsi)	37.5 (18.1)*	62.6 (28.1)	61.9 (22.2)	<0.001	25.22	0.241
Inhibitory control, incongruent mean RT (ms, Simon)	678.0 (166.6)*	474.8 (67.7)	489.8 (95.1)	<0.001	36.47	0.314

*significantly different from both other groups, children p’s < 0.001, adolescent and adults performance significantly different for eye-hand coordination spatial precision, *p* = 0.009, and proprioceptive variability, p = 0.025.

### Measures and Procedures

To assess sensorimotor and EF performance across this sample, age-appropriate valid and reliable tests were administered (i.e., no ceiling or floor effects for this age group). The sensorimotor tests included: Grasp Height task (anticipatory motor planning), Ipsilateral Remembered Joint Position Matching task (proprioceptive acuity), and Pursuit Rotor task (eye-hand coordination), as these tasks provide a comprehensive assessment of various upper limb sensorimotor functions. The cognitive tests measured EFs and included: the Tower of London (planning and problem-solving), Wisconsin Card Sorting task (cognitive flexibility), Corsi Block-Tapping task (visuospatial working memory), and Simon task (inhibitory control), as these tasks provide a comprehensive assessment of both core EFs, along with higher-level EF (cf. Diamond, 2013). The tests of EF were conducted using the Psychology Experiment Building Language (PEBL) software (see Piper et al., 2012; Mueller and Piper, 2014).

All participants were tested individually, and the experimenters and assistant were the same for all participants. Because prior research from our laboratory (cf. Stöckel and Hughes, 2016) has indicated that children are better able to sustain focus and concentration throughout the whole experiment when EF measures are sandwiched between sensorimotor tasks, the EF tasks were performed in the middle of the experimental session with the specific order in which the EF and sensorimotor tasks were performed randomized. Participants were given a 2 min break between tasks, and upon request. The testing session lasted between 60 and 90 min.

#### Sensorimotor Functions

The *Grasp Height Task* (Cohen and Rosenbaum, 2004; Seegelke and Hughes, 2015) was used to measure *anticipatory motor planning*. The shelving unit (92 cm × 30.5 cm × 213.4 cm) consisted of a home shelf located at 60% of the participant’s height, and five target shelves located at 40, 50, 60, 70, and 80% of the participant’s height. On the 60% height shelf an inner platform (45 cm × 15 cm, protruding 17 cm from the shelf) was positioned 40 cm from the side of the shelving unit for right hand dominant participants (10 cm from the side for left hand dominant participants), and served as the home platform. Another platform (45 cm × 15 cm) was adjustable and could be attached, 15 cm laterally from the home platform, on the five shelves and served as the target platform. The to-be manipulated object was a wooden dowel (54 cm in length, 2 cm in diameter) supported by a circular wooden base (10 cm in diameter and 5 cm high). Participants were asked to stand with their body centered in front of the home shelf (about 20 cm from the platform edge) and to use their dominant hand to grasp the object shaft and transport it from the home shelf to the target shelf, return their arm to their side, and then grasp and transport the plunger back to the home shelf. Participants performed four trials to each target platform height, yielding a total of 20 trials with platform heights fully randomized.

Kinematic data were collected using an eight-camera optical motion capture system (Vicon Motion Systems) that has a temporal and spatial resolution of 200 Hz and 1 mm, respectively. Retro-reflective markers (10 mm in diameter) were placed dorsally on the distal end of the second metacarpal (MCP), the styloid process of the radius, the styloid process of the ulna, and on the base of the object (OB). Grasp height was calculated as the vertical distance between MCP and OB, extracted from the first frame in which the object shaft was grasped at the home platform (home-to-target move) and the last frame for the target platform (target-to-home move). Participants showed motor planning if they grasped the shaft of the object at the same location or lower for the home-to-target moves than for the target-to-home moves for the platform at 80% of participant height, and at the same location or higher for the home-to-target moves than for the target-to-home moves for the platform at 40% of participant height (Wunsch et al., 2016). The primary outcome measure used is anticipatory motor planning score (number of successful trials from the highest shelf and lowest shelf, 0–8, **AMP**).

The *Ipsilateral Remembered Joint Position Matching Test* (Marini et al.,2017a,b) was used to measure proprioceptive acuity of the Flexion/Extension (FE) degree of freedom (DoF) of the wrist. Using a fully backdrivable robotic device (i.e., the WristBOT; see Marini et al., 2017b), participants sat in a height adjustable chair and placed their forearm in the robot support and grasped the robot handle. The torso and forearm of the participant was restrained with Velcro straps such that the wrist was aligned with the robot rotation axis and that the wrist was in the neutral anatomical configuration [0° of FE, Radial/Ulnar deviation (RUD), and Pronation/Supination (PS)] at the start of each trial. After being familiarized with the task, participants had their vision occluded using a pair of opaque glasses, and the task began. The start of each trial began with the WristBOT moving the wrist from the neutral start position to the target position [80% of the functional range of motion (ROM)]. At this point, the verbal cue “remember” was given to inform the participant to keep this specific wrist position in mind. After 3 s, the WristBOT brought the robot handle back to the start position (passive reaching phase). Subsequently, another verbal cue (“go”) marked the beginning of the next phase and cued the participant to move the robot handle so as to match the remembered target position (active reaching phase). The WristBOT then returned the robot handle to the start position (return phase). Participants completed 10 trials in each direction for a total of 20 trials. Wrist proprioceptive acuity was evaluated using the metrics matching error and variability. *Matching error* (an inverse measure of proprioceptive accuracy, **P_*ME*_**), where lower values are indicative of more accurate performance, was defined as the absolute value of the difference between the reference joint angle and the participant’s matching position. *Variability* (an inverse measure of proprioceptive consistency, **P_*Var*_**), where lower values are indicative of more precise performance, was defined as the standard deviation of matching position across all repetitions in each of the conditions. The matching error and variability values were averaged across all trials.

Additionally, the WristBOT was used to evaluate *eye-hand coordination* using a *Pursuit Rotor Task* (Koerth, 1922; Piper, 2011). In this task, a fire ball rotated around a circle (tangential speed of 21°/second, ideal horizontal and vertical diameter that corresponded to −25/+25 in FE and RUD), and participants were to move a rocket ship (a virtual representation of the WristBOT handle) so that it maintained contact with the fire ball to the best of the participant’s ability. Participants completed two practice trials, one in each direction (clockwise and counterclockwise). After the practice trials, each participant completed 8 trials in each direction, yielding a total of 16 trials. Eye-hand coordination performance was determined through *mean time-on-target* (percent of time, a measure of eye-hand coordination accuracy, **EH_*Acc*_**) and *linearity index* (the length of the rocket ship trajectory, 1 = perfect trajectory, a measure of eye-hand coordination spatial precision, **EH_*SP*_**) and were averaged across trials.

#### Executive Functions

The *Tower of London Task* (ToL) was administered to assess *response planning and problem-solving* abilities (Shallice, 1982; Anderson et al., 1996; Stöckel and Hughes, 2016) which are considered to be higher order EFs (Diamond, 2013). Participants were required to arrange a pile of disks from their original configuration, to match the configuration displayed at the top half of the computer screen for 12 different configurations. Participants were informed that they would only be able to move one disk at a time, could only move disks from the top of a pile and would not be able to move a disk onto a pile without space. Participants were also informed that they had a set number of moves that they needed to complete each trial in (two to five moves depending on the trial). The stimuli was based on the standard set of 12 problems (Shallice, 1982) that consists of three disks and constrained pile heights (1, 2, and 3). The primary outcome measure is ToL percent success (i.e., the percent of trials accurately solved in the minimum number of moves, **ToL**).

The 64-card version of the *Wisconsin Card Sorting Task* (WCST, Grant and Berg, 1948; Greve, 2001) was used to measure *cognitive flexibility* (also referred to as task switching). Participants were required to sort stimulus cards into four piles based on the card’s color, symbol or the number of symbols. However, they were not informed on how to classify the cards, only whether the classification was correct or incorrect based on the current sorting rule. Once the participant correctly sorted 10 cards following a specific classification, the sorting classification changed, and participants again needed to use the feedback provided (“correct” or “incorrect”) to figure out the new card sorting rule. Testing continued until all 64 cards were sorted. The primary outcome measure is percent correct (i.e., the percent of trials sorted according to the current rule, **WCST**).

The *Corsi Block-Tapping Test* (Corsi, 1972; Kessels et al., 2000; Stöckel and Hughes, 2016) was used to measure *visuospatial working memory* capacity. At the beginning of each trial participants could see blue-colored blocks arranged in a static spatial array on the computer screen with a black background. The blocks then changed color (i.e., from blue to yellow) in a specific sequence, after which the participant would then reproduce the sequence by clicking on the blocks with a mouse. The task began with three practice three-block sequences. Then the participant began the task with a two-block sequence and one block was added to the sequence after two trials at each sequence length (reaching a maximum length of nine blocks), if the participant was able to reproduce at least one of the two prior trials correctly. Two different trials were administered at each block span length. The test was discontinued when the participant was unable to successfully recall either trial of a given span or reached a block span of nine. Working memory was defined as the product of the number of correct trials and the maximum span length that resulted in the recall of at least one trial of a given span length (**Corsi**).

The *Simon Task* (Simon, 1969) was used to measure *inhibitory control*. Participants were instructed to press the left shift key (labeled in red) when a red dot appeared on the computer screen and to press the right shift key (labeled in blue) when a blue dot appeared on the screen as quickly and accurately as possible. They were provided standardized instructions and informed that the dots would appear to the right or left of the center of the screen. When a stimulus appeared on the same side as the response key, the trial was considered to be congruent, whereas the trial was considered incongruent when the stimulus appeared on the opposite side as the response key. The experiment consisted of 100 trials, comprised of 50 congruent and 50 incongruent trials, presented in a randomized order. Inhibition was measured using mean incongruent reaction time, measured in milliseconds (**Simon**).

### Data Processing and Statistical Analyses

Statistical analyses were run on data of 162 subjects. Consistent with data from the field of psychology (cf. Micceri, 1989), data was non-normal. Due to the non-normal distribution of data, the data were first transformed through the application of a rank-based inverse normal (RIN) transformation. This method has been shown to effectively transform skewed data into comparatively normal data without increasing the risk of Type I or Type II errors (Bishara and Hittner, 2012, 2015). Data were collapsed across sex, as systematic differences between males and females were not revealed in the initial stages of data analysis.

In the first step of the statistical analysis, one way ANOVAs were conducted on the normalized data separately for each dependent variable to confirm if significant differences in sensorimotor and EF performance exist between children (ages 8–12 years), adolescents (ages 13–17 years), and adults (ages 18–24 years). *Post-hoc* analysis was conducted for all sensorimotor and EF variables using Fisher’s LSD. Subsequently, to obtain a picture of the specific relations between sensorimotor functions and EFs, partial correlations (controlled for age) were performed between all normalized dependent variables, separately for each group (children, adolescents, adults). Finally, hierarchical multiple regression analyses were used to identify the sensorimotor and EF variables that predict motor skill behavior. This was done separately for each group. Due to the possible within-group effect of age, age was entered first into model 1 of the regression equations. Following model 1, model 2 included the sensorimotor and EF dependent variables found to have significant correlations to each of the five sensorimotor variables (anticipatory motor planning, proprioceptive matching error, proprioceptive variability, eye-hand coordination accuracy, and eye-hand coordination spatial precision).

## Results

### Age-Related Differences in Sensorimotor and Executive Function Performance

**FIGURE 1:**
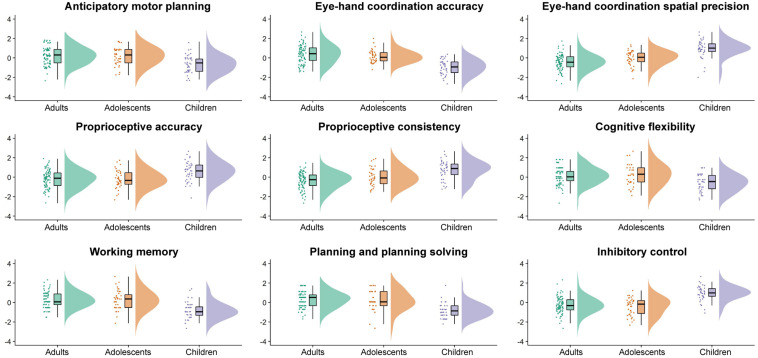
Raincloud plots showing the normalized data, normalized data distribution, and five summary statistics (i.e., normalized median, first quartile, third quartile, minimum, and maximum) for the adults (green), adolescents (orange), and children (purple) for the sensorimotor and executive function variables of interest.

Table 1 displays raw means and standard deviations for all sensorimotor and EF measures, with raincloud plots in Figure 1 depicting normalized data, data distribution, and summary statistics (i.e., median, first quartile, third quartile, minimum, and maximum) for all measures. As can be seen in Table 1, adolescents and adults outperformed children for all tested sensorimotor variables and EFs (*p*’s < 0.001). Adolescents performed similarly to adults for all measures except eye-hand coordination spatial precision (adolescents = 1.15, adults = 1.11, *p* = 0.009), and proprioceptive variability (adolescents = 4.75°, adults = 4.04°, *p* = 0.025).

### Age-Related Differences in Task-Specific Associations Between Sensorimotor and Executive Function Domains

Correlation analyses provided insights into how specific associations between sensorimotor and EFs differ between children, adolescents, and adults. In children (see Table 2), proprioceptive matching error and variability were positively correlated with one another (*r* = 0.680, *p* < 0.001). Correlations were also observed between measured EF variables. Specifically, planning and problem-solving was significantly correlated with both cognitive flexibility (*r* = 0.448, *p* = 0.004), and working memory (*r* = 0.353, *p* = 0.028). Finally, correlations were also observed between sensorimotor and EF measures, with cognitive flexibility significantly negatively correlated with proprioceptive matching error and variability (*r* = −0.352, and −0.382, respectively, both *p*’s < 0.05). Additionally, inhibitory control was found to be significantly negatively correlated with eye-hand coordination accuracy (*r* = −0.421, *p* = 0.008), and positively correlated with eye-hand coordination spatial precision (*r* = 0.318, *p* = 0.048). Lastly, working memory was found to be significantly correlated with eye-hand coordination accuracy (*r* = 0.351, *p* = 0.029).

**TABLE 2:** Partial correlations controlled for age between sensorimotor skill components and measures of executive functioning in children.

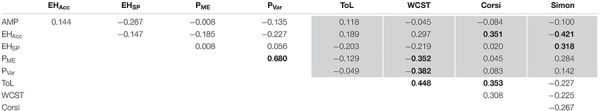

*Values in bold type represent significant correlations, *p* < 0.05. Gray boxes are the relationships between sensorimotor and EFs. AMP, Anticipatory motor planning; EH_*SP*_, Eye-hand coordination spatial precision; EH_*Acc*_, Eye-hand coordination accuracy; P_*M*__*E*_, Proprioceptive matching error; P_*Var*_, Proprioceptive variability; WCST, Cognitive flexibility; Corsi, Working memory; ToL, Planning and problem-solving; Simon, Inhibitory control.*

Correlational analyses for the adolescents are shown in Table 3. In adolescents, proprioceptive variability was positively correlated with proprioceptive matching error (*r* = 0.321, *p* = 0.049). Additionally, proprioceptive variability was negatively correlated with eye-hand coordination spatial precision (*r* = −0.437, *p* = 0.006) indicating that better proprioceptive variability is associated with worse eye-hand coordination spatial precision. No significant correlations were observed between EF measures, or sensorimotor measures and EF (all *p*’s > 0.05).

**TABLE 3:** Partial correlations controlled for age between sensorimotor skill components and measures of executive functioning in adolescents.

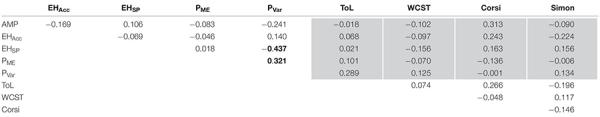

*Values in bold type represent significant correlations, *p* < 0.05. Gray boxes are the relationships between sensorimotor and EFs. AMP, Anticipatory motor planning; EH_*SP*_, Eye-hand coordination spatial precision; EH_*Acc*_, Eye-hand coordination accuracy; P_*M*__*E*_, Proprioceptive matching error; P_*Var*_, Proprioceptive variability; WCST, Cognitive flexibility; Corsi, Working memory; ToL, Planning and problem-solving; Simon, Inhibitory control.*

Correlational analyses for adults are shown in Table 4. In adults, proprioceptive matching error was positively correlated with proprioceptive variability (*r* = 0.296, *p* = 0.007) and negatively correlated with working memory (*r* = −0.471, *p* < 0.001). No other significant correlations were observed (all *p*’s > 0.05).

**TABLE 4:** Partial correlations controlled for age between sensorimotor skill components and measures of executive functioning in adults.

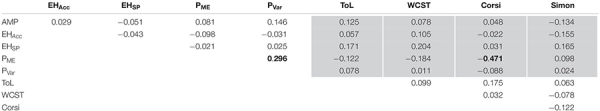

*Values in bold type represent significant correlations, *p* < 0.05. Gray boxes are the relationships between sensorimotor and EFs. AMP, Anticipatory motor planning; EH_*SP*_, Eye-hand coordination spatial precision; EH_*Acc*_, Eye-hand coordination accuracy; P_*M*__*E*_, Proprioceptive matching error; P_*Var*_, Proprioceptive variability; WCST, Cognitive flexibility; Corsi, Working memory; ToL, Planning and problem-solving; Simon, Inhibitory control.*

### Specific Processes Associated With Sensorimotor Performance

Hierarchical multiple regression analyses were used to identify the sensorimotor and EF variables that predict sensorimotor behavior, separately for each group. The results of each step in the regression analysis, individual standardized beta coefficients, and the associated significance can be found in Table 4, separately for each group (children, adolescents, adults).

With respect to children, regression analysis indicated that age (model 1) did predict eye-hand coordination accuracy (adjusted *R*^2^ = 0.188, *p* = 0.003), but the full model explained more of the variance of eye-hand coordination accuracy (adjusted *R*^2^ = 0.347, *p* < 0.001) beyond that of model 1 (*R*^2^ Δ = 0.189). With respect to the full model, inhibitory control (β = −0.395, *p* = 0.025) emerged as a unique contributor of eye-hand coordination accuracy. Eye-hand coordination spatial precision was significantly correlated with inhibitory control, yet regression analysis revealed that eye-hand coordination spatial precision could not be predicted by age alone (adjusted *R*^2^ = 0.014, *p* = 0.220) or the full model (adjusted *R*^2^ = 0.090, *p* = 0.066). For the variable proprioceptive matching error, regression analysis revealed that age did predict proprioceptive matching error (adjusted *R*^2^ = 0.104, *p* = 0.024), but the full model explained more of the variance of eye-hand coordination accuracy (adjusted *R*^2^ = 0.501, *p* < 0.001) beyond that of model 1 (*R*^2^ Δ = 0.412). With respect to the full model, proprioceptive variability (β = 0.642, *p* < 0.001) emerged as a unique contributor of proprioceptive accuracy. Finally, regression analysis of proprioceptive variability revealed that age alone did predict performance (adjusted *R*^2^ = 0.115, *p* = 0.018), but the full model explained more of the variance of proprioceptive variability (adjusted *R*^2^ = 0.418, *p* < 0.001) than model 1 (*R*^2^ change = 0.418). Specifically, proprioceptive matching error (β = 0.618, *p* < 0.001) emerged as a unique contributor of proprioceptive variability.

With respect to adolescents, regression analysis indicated that age alone did not predict eye-hand coordination spatial precision (adjusted *R*^2^ = 0.010, *p* = 0.246), but the full model did significantly predict eye-hand coordination spatial precision (adjusted *R*^2^ = 0.177, *p* = 0.011). With respect to the full model, proprioceptive variability (β = −0.431, *p* = 0.006) emerged as a unique contributor of eye-hand coordination spatial precision. For the variable proprioceptive matching error, regression analysis revealed that performance could not be predicted by age alone (adjusted *R*^2^ = 0.023, *p* = 0.176) or the full model (adjusted *R*^2^ = 0.099, *p* = 0.057). For proprioceptive variability, regression analysis indicated that though age alone did not predict performance (adjusted *R*^2^ = −0.021, *p* = 0.642), the full model did (adjusted *R*^2^ = 0.244, *p* = 0.005), with proprioceptive matching error (β = 0.336, *p* = 0.026), and eye-hand coordination spatial precision (β = −0.450, *p* = 0.004) emerging as unique contributors of proprioceptive variability.

With respect to adults, regression analysis indicated that age alone did not predict proprioceptive matching error (adjusted *R*^2^ = 0.002, *p* = 0.282), but the full model did significantly predict proprioceptive matching error (adjusted *R*^2^ = 0.271, *p* < 0.001). With respect to the full model, proprioceptive variability (β = 0.102, *p* = 0.009) and working memory (β = 0.112, *p* < 0.001) emerged as unique contributors of proprioceptive matching error. For the variable proprioceptive variability, regression analysis indicated that age alone did not predict performance (adjusted *R*^2^ = −0.009, *p* = 0.603), but that the full model did (adjusted *R*^2^ = 0.068, *p* = 0.022), with proprioceptive matching error (β = 0.298, *p* = 0.007) emerging as a unique contributor of proprioceptive variability.

## Discussion

The specific aims of the present study were (1) to assess how children, adolescents, and adults differ in their performance of sensorimotor functions and EFs (with adults representing mature performance) and (2) to explore the specific links between sensorimotor functions and EFs at different periods of development. Consistent with previous research (Luciana et al., 2009; Wunsch et al., 2016; Marini et al., 2017b; Stuhr et al., 2020), the level of performance was better in adults and adolescents compared to children. Moreover, with the exception of two measures of sensorimotor functioning (i.e., eye-hand coordination spatial precision and proprioceptive variability), adult and adolescent performance was not statistically different.

Taken together, these findings suggest that all tested EFs reach maturation during adolescence (Farrell Pagulayan et al., 2006; Luciana et al., 2009; Dajani and Uddin, 2015). This finding aligns with previous neuropsychological research demonstrating that the brain areas responsible for high level cognitive functioning [prefrontal cortex (Luna et al., 2010) and cerebellum (Romero et al., 2021)] develop over an extended time frame and do not reach maturity (e.g., adult like neural activation levels during cognitive tasks due to increased myelination and changes in white and gray matter density) until adolescence. In contrast, the time period at which sensorimotor functions reach maturity differ, with some functions reaching maturation in adolescence (i.e., anticipatory motor planning, eye-hand coordination accuracy, proprioceptive accuracy), and others continue to develop into early adulthood (i.e., eye-hand coordination spatial precision, proprioceptive consistency; Leversen et al., 2012; Sigmundsson et al., 2016; Payne and Isaacs, 2017). It has been exhibited that sensorimotor accuracy develops before consistency (Schmidt et al., 2018), and as such, we hypothesize that the two measures that continue to develop into early adulthood exhibit a longer developmental trajectory because they are measures of sensorimotor consistency.

Regarding the specific links between sensorimotor functions and EFs, it was found that the relations between these functions differed between the groups. Specifically, in the child group, cognitive flexibility was associated with both measures of proprioception, inhibitory control was associated with both measures of eye-hand coordination, and working memory was associated with eye-hand coordination accuracy (see Table 5). We postulate that these specific interrelations arise from the cognitive requirements that underlie successful sensorimotor performance, which are still developing in children between 8 and 12 years of age. For example, successful eye-hand coordination requires the coordination of multiple sensory systems (i.e., visual, proprioceptive, vestibular), and control over multiple joints (i.e., shoulder, elbow, wrist; Durkina et al., 1995). Additionally, while an individual performs a task that requires eye-hand coordination, they must be able to refrain from sudden corrective movements, and maintain a mental representation of the task’s goal in order to update motor commands in anticipation and reaction to movement errors. As such, it is not surprising that both inhibitory control and working memory support eye-hand coordination performance in childhood.

**TABLE 5:** Hierarchical multiple regression results.

	Model		Standardized β	*P*	Adjusted *R*^2^	*R*^2^Δ	*P*
**Children**							
Eye-hand coordination accuracy, mean time-on-target (%, EH_Acc_)	Model 1	Age	0.457	**0.003**	0.188	0.209	**0.003**
	Model 2	Age	0.133	0.422	0.347	0.189	**<0.001**
		Corsi	0.243	0.098			
		Simon	−0.395	**0.025**			
Eye-hand coordination spatial precision, linearity index (EH_SP_)	Model 1	Age	−0.198	0.220	0.014	0.039	0.220
	Model 2	Age	0.039	0.838	0.090	0.097	0.066
		Simon	0.392	0.048			
Proprioceptive acuity, matching error (°, P_ME_)	Model 1	Age	−0.356	**0.024**	0.104	0.127	**0.024**
	Model 2	Age	−0.088	0.476	0.501	0.412	**<0.001**
		P_Var_	0.642	**<0.001**			
		WCST	−0.106	0.412			
Proprioceptive acuity, variability (°, P_Var_)	Model 1	Age	−0.372	**0.018**	0.115	0.138	**0.018**
	Model 2	Age	−0.108	0.374	0.519	0.418	**<0.001**
		P_ME_	0.618	**<0.001**			
		WCST	−0.157	0.212			
**Adolescents**							
Eye-hand coordination spatial precision, linearity index (EH_SP_)	Model 1	Age	−0.190	0.246	0.010	0.036	0.246
	Model 2	Age	−0.223	0.139	0.177	0.184	**0.011**
		P_Var_	−0.431	**0.006**			
Proprioceptive acuity, matching error (°, P_ME_)	Model 1	Age	−0.221	0.176	0.023	0.049	0.176
	Model 2	Age	−0.197	0.210	0.099	0.098	0.057
		P_Var_	0.314	0.049			
Proprioceptive acuity, variability (°, P_Var_)	Model 1	Age	−0.077	0.642	−0.021	0.006	0.642
	Model 2	Age	−0.088	0.553	0.244	0.298	**0.005**
		EH_SP_	−0.450	**0.004**			
		P_ME_	0.336	**0.026**			
**Adults**							
Proprioceptive acuity, matching error (°, P_ME_)	Model 1	Age	0.119	0.282	0.002	0.014	0.282
	Model 2	Age	0.200	**0.042**	0.271	0.283	**<0.001**
		P_Var_	0.256	**0.009**			
		Corsi	−0.455	**<0.001**			
Proprioceptive acuity, variability (°, P_Var_)	Model 1	Age	0.058	0.603	−0.009	0.003	0.603
	Model 2	Age	0.022	0.836	0.068	0.088	**0.022**
		P_ME_	0.298	**0.007**			

*Bold-typed values represent significant, *p* < 0.05. AMP, Anticipatory motor planning; EH_SP_, Eye-hand coordination spatial precision; EH_*Acc*_, Eye-hand coordination accuracy; P_*ME*_, Proprioceptive matching error; P_*Var*_, Proprioceptive variability; WCST, Cognitive flexibility; Corsi, Working memory; ToL, Planning and problem-solving; Simon, Inhibitory control.*

This hypothesis is supported by the data of adolescents, where no relations were found between sensorimotor and EF performance, demonstrating that throughout the course of development, sensorimotor functions become less reliant on EFs to support successful sensorimotor performance. There is emerging evidence that the relations between sensorimotor functions and EFs is stronger in children (under the age of 13 years) compared to adolescents (13 years of age and older; Rigoli et al., 2012; Stöckel and Hughes, 2016). These behavioral observations are supported by findings indicating that sensorimotor functions and EFs develop at a similar rate in childhood, but begin to take more independent and separate developmental trajectories by adolescence (van der Fels et al., 2015). Taken together, these findings highlight the important role EFs play in supporting sensorimotor functioning in childhood, and how these relations change during adolescence.

With respect to the relations between sensorimotor functions, a group specific relation was found between eye-hand coordination spatial precision and proprioceptive variability in the adolescent group. Specifically, eye-hand coordination spatial precision explained a unique portion of proprioceptive variability (see Table 5), with results indicating that adolescents with better eye-hand coordination spatial precision exhibited worse proprioceptive variability. This finding may be the result of the sensory mode of control adolescents prioritize when executing specific actions (Prochazka and Ellaway, 2012), whereby adolescents tend to rely on one sense more and simultaneously neglect the other (Viel et al., 2009). However, as these systems develop further, individuals are more able to balance multiple sensory modes. Given the emerging evidence that adolescents experience sensorimotor regression in specific motor control aspects (e.g., intersegmental/interlimb coordination, neuromuscular control; Quatman-Yates et al., 2012), it is possible that this phenomenon could lead to an inverse relation between the performance of eye-hand coordination spatial precision and proprioceptive variability during adolescence.

The present study provides new evidence about the interaction between sensorimotor functions and EFs at different stages of development, and how these differ from young adults (representing upper bound performance). Even so, there are some limitations to this study. First, data collection was cut short due to the beginning of the SARS-CoV-2 pandemic and subsequent continuous lockdowns in the region where data was collected (all data was collected prior to the pandemic onset). Though a more thorough analysis of the changes to, and relationships between sensorimotor functions and EFs could not be made, to our knowledge, no previous study has examined the relationship between sensorimotor functions and EFs in child, adolescent, and adults groups. Another limitation may have been the task used to measure anticipatory motor planning, as performance in all three groups was not associated with any other measured sensorimotor function or EF. It may have been that the continuous nature of the task (grasping anywhere along the object handle) made it a less sensitive measure of anticipatory motor planning than other tasks such as the unimanual or bimanual bar transport tasks, where previous studies have found associations between anticipatory motor planning and EF performance at different stages in the lifespan (Stöckel and Hughes, 2016; Stöckel et al., 2017).

Limitations notwithstanding, the findings from the present study contribute to existing literature regarding the development of sensorimotor functions and EFs, and how the relations between these functions differ at varying stages in life. The present results indicate that EFs reach maturation during adolescence, but that sensorimotor consistency continues to develop into adulthood. Additionally, results demonstrate that the interrelations between sensorimotor functions and EFs are dynamic with EF performance influencing children’s sensorimotor performance, but not adolescent’s sensorimotor performance. The next step in this line of study is to analyze the moderating effects that environmental factors (e.g., participation in extracurricular activities, socioeconomic status, video game/technology use) have on sensorimotor and EF performance and development. This field of study would also benefit from the use of longitudinal designs, over the course of several years, as this is necessary in order to draw first conclusions about the rate and shape of development, and may inform on the most relevant time points in which to provide interventions. From an applied perspective, the existing research indicates that children demonstrating sensorimotor delays may benefit from EF interventions, as the relationships between sensorimotor functions and EFs are prevalent during this stage in life.

## Data Availability Statement

The raw data supporting the conclusions of this article will be made available by the authors, without undue reservation.

## Ethics Statement

The studies involving human participants were reviewed and approved by the San Francisco State University Institutional Review Board. Written informed consent to participate in this study was provided by the participants (age 18 years and older), or participants’ legal guardian/next of kin (ages 8 to 17 years).

## Author Contributions

CG-M, TS, and CH designed the experiment, formulated the experimental question, performed the data analysis and statistics, and drafted the manuscript. CG-M, MS, and CH performed the data collection. All authors approved the final version.

## Conflict of Interest

The authors declare that the research was conducted in the absence of any commercial or financial relationships that could be construed as a potential conflict of interest.

## Publisher’s Note

All claims expressed in this article are solely those of the authors and do not necessarily represent those of their affiliated organizations, or those of the publisher, the editors and the reviewers. Any product that may be evaluated in this article, or claim that may be made by its manufacturer, is not guaranteed or endorsed by the publisher.
